# No added diagnostic value of non-phosphorylated tau fraction (p-tau_rel_) in CSF as a biomarker for differential dementia diagnosis

**DOI:** 10.1186/s13195-017-0275-5

**Published:** 2017-07-14

**Authors:** Joery Goossens, Maria Bjerke, Hanne Struyfs, Ellis Niemantsverdriet, Charisse Somers, Tobi Van den Bossche, Sara Van Mossevelde, Bart De Vil, Anne Sieben, Jean-Jacques Martin, Patrick Cras, Johan Goeman, Peter Paul De Deyn, Christine Van Broeckhoven, Julie van der Zee, Sebastiaan Engelborghs

**Affiliations:** 1 Reference Center for Biological Markers of Dementia, Laboratory of Neurochemistry and Behavior, Institute Born-Bunge, University of Antwerp, Universiteitsplein 1, 2610 Wilrijk, Belgium; 2 Neurodegenerative Brain Diseases Group, Department of Molecular Genetics, VIB, Universiteitsplein 1, 2610 Wilrijk, Belgium; 3 Laboratory of Neurogenetics, Institute Born-Bunge, University of Antwerp, Universiteitsplein 1, 2610 Wilrijk, Belgium; 4 Department of Neurology and Memory Clinic, Hospital Network Antwerp (ZNA) Middelheim and Hoge Beuken, 2660 Antwerpen, Belgium; 5 Department of Neurology, Antwerp University Hospital, Wilrijkstraat 10, 2650 Edegem, Belgium; 6 Laboratory of Neurology, Translational Neurosciences, University of Antwerp, Universiteitsplein 1, 2610 Wilrijk, Belgium; 7 Laboratory of Neurobiology, Institute Born-Bunge, University of Antwerp, Universiteitsplein 1, 2610 Wilrijk, Belgium; 8 Biobank, Institute Born-Bunge, University of Antwerp, Universiteitsplein 1, 2610 Wilrijk, Belgium

**Keywords:** Alzheimer’s disease, Differential diagnosis, Cerebrospinal fluid, Biomarkers, Tau, Frontotemporal lobar degeneration, Dementia with Lewy bodies, Creutzfeldt-Jakob disease

## Abstract

**Background:**

The Alzheimer’s disease (AD) cerebrospinal fluid (CSF) biomarkers Aβ_1–42_, t-tau, and p-tau_181_ overlap with other diseases. New tau modifications or epitopes, such as the non-phosphorylated tau fraction (p-tau_rel_), may improve differential dementia diagnosis. The goal of this study is to investigate if p-tau_rel_ can improve the diagnostic performance of the AD CSF biomarker panel for differential dementia diagnosis.

**Methods:**

The study population consisted of 45 AD, 45 frontotemporal lobar degeneration (FTLD), 45 dementia with Lewy bodies (DLB), and 21 Creutzfeldt-Jakob disease (CJD) patients, and 20 cognitively healthy controls. A substantial subset of the patients was pathology-confirmed. CSF levels of Aβ_1–42_, t-tau, p-tau_181_, and p-tau_rel_ were determined with commercially available single-analyte enzyme-linked immunosorbent assay (ELISA) kits. Diagnostic performance was evaluated by receiver operating characteristic (ROC) curve analyses, and area under the curve (AUC) values were compared using DeLong tests.

**Results:**

The diagnostic performance of single markers as well as biomarker ratios was determined for each pairwise comparison of different dementia groups and controls. The addition of p-tau_rel_ to the AD biomarker panel decreased its diagnostic performance when discriminating non-AD, FTLD, and DLB from AD. As a single marker, p-tau_rel_ increased the diagnostic performance for CJD. No significant difference was found in AUC values with the addition of p-tau_rel_ when differentiating between AD or non-AD dementias and controls.

**Conclusions:**

The addition of p-tau_rel_ to the AD CSF biomarker panel failed to improve differentiation between AD and non-AD dementias.

**Electronic supplementary material:**

The online version of this article (doi:10.1186/s13195-017-0275-5) contains supplementary material, which is available to authorized users.

## Background

**Fig. 1:**
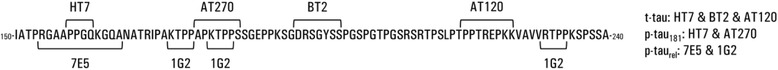
Epitopes of different tau assays. Binding sites of antibodies making up total tau (*t-tau*), tau protein phosphorylated at threonine 181 (*p-tau*_*181*_), and non-phosphorylated tau fraction *(p-tau*_*rel*_ ) assays. Binding of antibody AT270 requires phosphorylation of threonine (T), while binding of antibody 1G2 requires threonine (T) to be not phosphorylated

Cerebrospinal fluid (CSF) biomarkers are being used to improve the clinical diagnostic accuracy of Alzheimer’s disease (AD) [1, 2]. Established markers include levels of amyloid-beta of 42 amino acids (Aβ_1–42_), total tau protein (t-tau), and phosphorylated tau protein (p-tau) [3]. The most studied isoform of p-tau is that with phosphorylation at threonine 181 (p-tau_181_), while there are other phosphorylated tau epitopes that have not been thoroughly investigated for their clinical utility such as serine 199 and threonine 231 [4]. Although studies on large neuropathology case series suggest that tau pathology precedes amyloid-β plaque pathology [5], the opposite holds true for the detectable biomarker counterparts in CSF [6]. Thus, there is a discrepancy between neuropathology and detectable clinical biomarker findings [7]. Furthermore, since the current biomarkers are already changed in the mild cognitive impairment (MCI) stage and remain stable during the clinical course, they cannot be used as prognostic markers. As single markers they are not completely specific for AD given that there is a slight overlap with other neurodegenerative diseases, hampering their usefulness as differential diagnostic markers [8, 9]. Indeed, it appears that current assay setups are not sensitive enough to detect, for example, disease-specific changes in tau processing. Together, these limitations have spurred the search for new tau modifications or epitopes that may improve early and differential diagnosis. Recently, a novel assay became available that can detect the non-phosphorylated tau fraction (p-tau_rel_) in CSF [10]. This assay was developed to specifically detect tau protein with no phosphorylation at epitopes threonine T175, T181, and T231 (Fig. 1; [10]). It was found that p-tau_rel_ is significantly higher in an AD/MCI cohort in comparison with controls, while differentiation between MCI and AD was not possible [10]. The assay was also proposed to be helpful when differentiating between AD and other dementias, particularly tauopathies. The goal of this study was thus to investigate whether p-tau_rel_ can improve the diagnostic performance of the AD CSF biomarker panel for differential dementia diagnosis, comparing AD with a variety of non-AD dementias.

## Methods

### Study cohort

The study population consisted of clinically diagnosed AD patients (*n* = 45), definite frontotemporal lobar degeneration (FTLD) patients (*n* = 45, of which 33 were FTLD-TDP, 10 FTLD-tau, and 2 FTLD-UPS), both clinical and definite Lewy body dementia (DLB) patients (*n* = 45, of which 19 definite), definite Creutzfeldt-Jakob disease (CJD) patients (*n* = 21), and cognitively healthy controls (*n* = 20). To ensure high diagnostic accuracy in the clinically diagnosed patients, only patients with clinical follow-up were included. All CSF samples were selected from the Institute Born-Bunge Biobank, Antwerp, Belgium [1, 11]. Patient and control-associated information included in the statistical analyses consisted of gender, age at CSF sampling, and age at death (if applicable). This study was approved by the ethics committee of UAntwerp, Antwerp, Belgium. Informed consent was obtained from all subjects.

Clinical diagnosis of AD was based on IWG-2 criteria and included the AD CSF biomarker panel (Aβ_1–42_ (cut-off: 638.5 pg/ml), t-tau (cut-off: 296.5 pg/ml), and p-tau_181_ (cut-off: 56.5 pg/ml)) [2, 11, 12]. Definite FTLD diagnosis was defined by genetic carrier status (*n* = 30) and/or postmortem neuropathological confirmation of brain pathology (*n* = 28) [13, 14]. Clinical and neuropathological diagnoses of DLB were based on standard diagnostic criteria [15]. All patients in the CJD subgroup met the clinical diagnostic criteria, including 14-3-3 positivity [16], and were also RT-QuiC positive proving prion-pathology [17]. Moreover, 10 CJD patients had neuropathological confirmation [18]. The control group consisted of subjects with no history of neurological and psychiatric disorders or organic disease involving the central nervous system, as established by extensive clinical examination (patients with polyneuropathy: *n* = 8; patients with subjective physical complaints: *n* = 12).

### Biomarker analysis

Lumbar puncture, and CSF sampling and handling was performed according to a standard protocol [11, 19]. All samples were stored in polypropylene vials at –80°C until analysis.

CSF levels of Aβ_1–42_, t-tau, p-tau_181_, and p-tau_rel_ were determined with commercially available single-analyte enzyme-linked immunosorbent assay (ELISA) kits (one kit lot each), strictly following the manufacturer’s instructions (INNOTEST β-Amyloid(_1–42_), INNOTEST hTau-Ag, and INNOTEST Phospho-Tau(_181P_) from Fujirebio Europe, Belgium; pTAU rel ELISA Kit from AJ Roboscreen, IBL International GmbH, Germany). All samples were run in duplicate and blinded for diagnosis. Intra-assay coefficient of variation (CV) was below 10% and inter-assay CV below 15% for all analytes.

### Statistical analysis

Statistical testing was performed using IBM SPSS Statistics 23, GraphPad Prism 6, and MedCalc. Kruskall-Wallis analyses with post-hoc Dunn’s multiple comparisons tests were performed to describe our patient cohort and compare biomarker levels between groups. Pairwise comparisons were only performed between controls and different dementia groups and between AD and different non-AD dementia groups. Categorical variables were analyzed with a Chi-square test. Spearman’s rho was calculated to determine correlations. Receiver operating characteristic (ROC) curve analyses were used to obtain area under the curve (AUC) values for differentiation between groups. AUC values were compared using DeLong tests. Maximal sum of sensitivity and specificity (maximized Youden’s index) was calculated to determine cut-off values. For all analyses, *p* values below 0.05 were considered statistically significant.

## Results

**Fig. 2:**
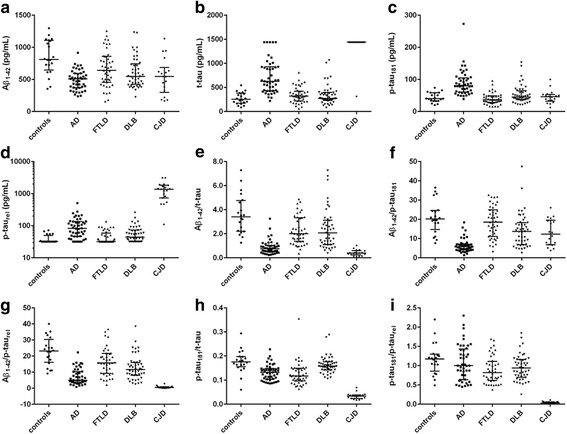
Dot plots of individual markers and ratios. Dot plots showing individual biomarker levels in each subgroup. **a** amyloid-beta of 42 amino acids (*Aβ*_*1–42*_); **b** total tau protein (*t-tau*); **c** tau protein phosphorylated at threonine 181(*p-tau*_*181*_); **d** non-phosphorylated tau fraction (*p-tau*_*rel*_); **e** Aβ_1–42_/t-tau; **f** Aβ_1–42_/p-tau_181_; **g** Aβ_1–42_/p-tau_rel_; **h** p-tau_181_/t-tau; **i** p-tau_181_/p-tau_rel_. Lines indicate median with interquartile range. *AD* Alzheimer’s disease, *CJD* Creutzfeldt-Jakob disease, *DLB* dementia with Lewy bodies, *FTLD* frontotemporal lobar degeneration

Demographic, clinical, and biomarker data of all groups are summarized in Table 1 and Fig. 2. Patient cohorts were not matched for age and gender but there was no observable effect of these parameters on biomarker levels (data not shown). Two FTLD patients and two CJD patients were excluded from statistical analysis as all their biomarker values were below the respective limits of detection, probably related to preanalytical factors. Concentration of t-tau was above the detection limit in 5/45 AD and 17/19 CJD cases and below the detection limit in 1/43 FTLD cases; Aβ_1–42_ was below the detection limit in 1/19 CJD cases only; p-tau_181_ was below the detection limit in 3/43 FTLD cases and 1/19 CJD cases; p-tau_rel_ was below the detection limit in 13/20 controls, 4/45 AD, 21/43 FTLD, and 13/45 DLB cases. As dilution of samples is not recommended for the three INNOTEST assays, out-of-range biomarker values were set to the lowest/highest detection point ±20%, and this value was used in statistical analyses. Due to the high number of samples with out-of-range biomarker values (especially the case for p-tau_rel_, see Discussion), non-parametric statistical analyses were performed.

**Table 1:** Demographic, clinical, and biomarker data

	Controls	AD	FTLD	DLB	CJD	*p* value
Gender (% male/female) (*n*)	55/45 (20)	49/51 (45)	51/49 (45)	71/39 (45)	33/67 (21)	**0.049**
Age at CSF sampling (years)	69.4 (61.5–74.7)	71.2 (66.7–79.2)	63.6 (55.1–71.7)	75.5 (71.2–81.2)	67.2 (57.4–76.4)	**<0.001** ^**e**^
MMSE (0–30) (*n*)	NA*	20 (15–25) (42)	21 (15–25) (29)	19 (16–23) (38)	NA	0.54
Aβ_1–42_ (pg/mL)	812 (646–1108)	509 (372–594)	641 (457–858)	547 (423–744)	545 (300–686)	**<0.001** ^**a,c,d,e**^
t-tau (pg/mL)	257 (173–381)	627 (429–928)	320 (219–420)	272 (232–398)	>1440^$^	**<0.001** ^**a,d,e,f**^
p-tau_181_ (pg/mL)	40.3 (32.9–58.6)	80.0 (60.5–105.0)	36.7 (28.3–49.0)	45.0 (39.8–64.7)	46.0 (32.2–53.4)	**<0.001** ^**a,e,f,g**^
p-tau_rel_ (pg/mL)	32.0 (32.0–49.7)	82.7 (46.6–135.7)	32.0 (32.0–59.0)	44.3 (32.0–73.8)	1375 (738–1820)	**<0.001** ^**a,d,e,f,g**^
Aβ_1–42_/t-tau	3.40 (2.20–4.76)	0.75 (0.50–1.02)	2.00 (1.36–3.33)	2.08 (1.10–3.13)	0.39 (0.24–0.60)	**<0.001** ^**a,d,e,f**^
Aβ_1–42_/p-tau_181_	20.2 (14.7–24.7)	5.8 (4.1–7.1)	18.5 (11.1–25.0)	13.7 (6.8–18.3)	12.3 (6.8–19.6)	**<0.001** ^**a,c,e,f,g**^
Aβ_1–42_/p-tau_rel_	23.1 (16.2–30.3)	5.0 (3.5–10.3)	15.8 (9.2–21.6)	11.6 (8.0–16.0)	0.4 (0.2–0.9)	**<0.001** ^**a,c,d,e,f,g**^
p-tau_181_/t-tau	0.176 (0.156–0.197)	0.132 (0.104–0.149)	0.117 (0.097–0.149)	0.158 (0.143–0.177)	0.033 (0.024–0.038)	**<0.001** ^**a,b,d,f,g**^
p-tau_181_/p-tau_rel_	1.17 (0.85–1.30)	1.00 (0.64–1.43)	0.82 (0.61–1.11)	0.94 (0.70–1.16)	0.03 (0.03–0.06)	**<0.001** ^**d,g**^
*APOE*ε4 carriers (%) (*n*)	40.0 (5)	61.1 (36)	32.1 (28)	33.3 (33)	NA	0.059

Values are presented as median (interquartile range), percentage (%) or number (*n*)

Gender distribution was compared by Chi-square test

Significant differences in clinical data and biomarker levels were determined by Kruskal-Wallis with post-hoc Dunn’s correction: ^a^ controls vs. AD; ^b^ controls vs. FTLD; ^c^ controls vs. DLB; ^d^ controls vs. CJD; ^e^ AD vs. FTLD; ^f^ AD vs. DLB; ^g^ AD vs. CJD

Age at CSF sampling was also significantly different for FTLD vs. DLB and CJD vs. DLB

Statistically significant *p* values (<0.05) are marked in bold

*MMSE only performed when clinically relevant (*n* = 3), no score <27

^$^Most CJD patients had t-tau values above the detection limit, which were set to highest point of the standard curve +20%

*Aβ*_*1–42*_ amyloid-beta of 42 amino acids, *AD* Alzheimer’s disease, *CJD* Creutzfeldt-Jakob disease, *CSF* cerebrospinal fluid, *DLB* dementia with Lewy bodies, *FTLD* frontotemporal lobar degeneration, *MMSE* Mini-Mental State Examination, *NA* not available, *p-tau*_*181*_ tau protein phosphorylated at threonine 181, *p-tau*_*rel*_ non-phosphorylated tau fraction, *t-tau* total tau protein

AD patients had lower levels of Aβ_1–42_ (median (range), 509 (372–594) pg/mL) and higher t-tau (627 (429–928) pg/mL), p-tau_181_ (80.0 (60.5–105.0) pg/mL), and p-tau_rel_ (82.7 (46.6–135.7) pg/mL) levels than controls and non-AD patients, with the exception of CJD patients who had the highest t-tau and p-tau_rel_ levels (Table 1). In all groups, the three different markers for tau significantly correlated with each other, with the correlation between t-tau and p-tau_rel_ being markedly stronger (Spearman’s rho between 0.70–0.84) than the correlation between p-tau_181_ and p-tau_rel_ in every subgroup (Spearman’s rho between 0.47–0.77; Additional file 1). Correlations with t-tau could not be calculated for CJD, given that 17/19 samples had a fixed t-tau value (i.e., the real value was above the detection limit of the assay).

To evaluate the diagnostic performance of p-tau_rel_ in comparison to the AD CSF biomarker panel, the single established AD marker with the highest AUC value was compared with that of p-tau_rel_. For example, the single marker which had the most diagnostic power to differentiate between AD and non-AD dementias was p-tau_181_, achieving an AUC of 0.883 (sensitivity: 88.9%; specificity: 76.4%). On the other hand, the AUC of p-tau_rel_ was significantly lower at 0.619 (sensitivity: 80.0%; specificity: 48.6%). Next to single markers, the AD biomarker ratio with the highest AUC value was also compared with its equivalent using p-tau_rel_. Continuing the example of AD versus non-AD differentiation, the best ratio with an AUC of 0.860 (sensitivity: 84.4%; specificity: 78.3%) was Aβ_1–42_/p-tau_181_. This value was thus compared to that of the Aβ_1–42_/p-tau_rel_ ratio, which was also significantly lower at 0.657 (sensitivity: 68.9%: specificity: 67.0%). Pairwise comparison between ROC curves with the highest AUC values, for both single markers and biomarker ratios, was performed for each AD versus non-AD dementia group (FTLD, DLB, and CJD), as well as for each different dementia group versus controls (Table 2). A complete overview of all ROC curve analyses can be found in Additional file 1.

**Table 2:** Diagnostic performance of the AD CSF biomarker panel compared to p-tau_rel_

	AD panel		p-tau_rel_	Aβ_1–42_/p-tau_rel_	p-tau_181_/p-tau_rel_
AD vs. non-AD	p-tau_181_	0.883	0.619^**$**^		
	Aβ_1–42_/p-tau_181_	0.860		0.657^**$**^	
AD vs. FTLD	p-tau_181_	0.933	0.799^**$**^		
	Aβ_1–42_/p-tau_181_	0.920		0.846^**$**^	
AD vs. DLB	t-tau	0.863	0.706^**$**^		
	Aβ_1–42_/t-tau	0.855		0.747^**$**^	
AD vs. CJD	t-tau	0.896	0.978*		
	p-tau_181_/t-tau	1.000			1.000
AD vs. controls	t-tau	0.926	0.865		
	Aβ_1–42_/t-tau	0.976		0.949	
FTLD vs. controls	Aβ_1–42_	0.679	0.588		
	p-tau_181_/t-tau	0.827			0.695
DLB vs. controls	Aβ_1–42_	0.747	0.717		
	Aβ_1–42_/p-tau_181_	0.739		0.815	
CJD vs. controls	t-tau	0.978	1.000		
	Aβ_1–42_/t-tau	1.000		1.000	

The third column contains AUC values for the established single markers and biomarker ratios with the highest diagnostic value

Adjacent columns show AUC values of p-tau_rel_ and the equivalent ratio using p-tau_rel_

Significant differences between AUC values (DeLong tests, *p* < 0.05) are marked by an asterisk (*) when diagnostic power is significantly higher with addition of p-tau_rel_, and by a dollar sign (^**$**^) when diagnostic power is significantly lower with addition of p-tau_rel_

*Aβ*_*1–42*_ amyloid-beta of 42 amino acids, *AD* Alzheimer’s disease, *CJD* Creutzfeldt-Jakob disease, *CSF* cerebrospinal fluid, *DLB* dementia with Lewy bodies, *FTLD* frontotemporal lobar degeneration, *p-tau*_*181*_ tau protein phosphorylated at threonine 181, *p-tau*_*rel*_ non-phosphorylated tau fraction, *t-tau* total tau protein

## Discussion

This study aimed to evaluate the diagnostic value of the non-phosphorylated tau fraction in comparison to that of the established AD biomarker panel for the differentiation between AD and non-AD dementias.

### AD versus non-AD dementias

When looking at the established AD biomarker panel, the differentiation between AD and non-AD dementias remained suboptimal, and our results confirm previous research in autopsy-confirmed patients that reported p-tau_181_ to be the most fundamental component of the AD CSF biomarker panel, showing the highest differential discriminatory power [8]. Given that p-tau_rel_ is thought to inversely represent hyperphosphorylated tau [10], a comparable performance to p-tau_181_ could be expected. However, our results showed that the use of p-tau_rel_ as a single marker resulted in a significantly lower AUC value for the comparison between AD and non-AD, and also the combination of p-tau_rel_ together with the three established AD biomarkers did not improve the differential diagnostic accuracy for these dementia groups.

As tau-positive inclusions can also be found as a distinct primary pathology in a significant subgroup of FTLD [20], and pathological hyperphosphorylation of tau might be different to that in AD, there was a diagnostic potential for p-tau_rel_ to be of value in the differentiation between AD and FTLD. This was, however, not the case, as both the AUC value of p-tau_rel_ alone and that of the Aβ_1–42_/p-tau_rel_ ratio were significantly lower than those of the equivalent established AD biomarkers. It should be noted that the difference in AUC values for the biomarker ratio lost significance when *APOE*ε4 carrier status was taken into account, as this had an effect on Aβ_1–42_ values in FTLD patients (*p* = 0.036). Finally, there was no significant difference for any single biomarker or biomarker ratio between FTLD-tau-positive and FTLD-tau-negative subgroups (data not shown).

Patients with DLB can often present with an AD-like co-pathology, thus limiting the value of the established AD biomarkers [21]. Nonetheless, reasonable AUC values were obtained when comparing AD and DLB, corroborating our patient selection of DLB with almost half having a neuropathological confirmation. While there is no direct reason to assume different results by using p-tau_rel_, there was a significant decrease in AUC values for both single marker and biomarker ratio ROC analyses.

One interesting result was found for p-tau_rel_ as a single marker in the comparison of AD and CJD, obtaining a significantly higher AUC value than that of t-tau. CJD is typically characterized by very high concentrations of t-tau, reflecting the extensive neurodegeneration, and the protein can act as surrogate marker for the disease [22, 23]. On the other hand, the p-tau_181_/p-tau_rel_ ratio did not perform better than the p-tau_181_/t-tau ratio. This suggests that the (relatively) low AUC value for t-tau in the AD vs. CJD differentiation is most likely due to the limited detection range of the used t-tau assay, as true t-tau values could not be obtained for 17/19 CJD cases. As such, p-tau_rel_ is not necessarily a better biomarker in CJD, but the assay gives more accurate results in this cohort.

### AD and non-AD dementias versus controls

Within the established AD biomarker panel, an excellent AUC value was found for the ROC curve between AD and controls, confirming the sensitivity of these biomarkers for AD detection. The AUC values obtained with the addition of p-tau_rel_ were not significantly different for either single marker or biomarker ratio. However, the observed AUC value for p-tau_rel_ when differentiating AD from controls (0.865; sensitivity: 88.9%; specificity: 75.0%) was lower than that obtained by Lewczuk et al. (0.976; sensitivity: 94.8%; specificity: 97.6%) [10]. The decreased specificity in this study can be explained by the fact that many control subjects had p-tau_rel_ concentrations below the detection limit (see below). While this issue should be acknowledged when interpreting all ROC analyses including controls, we believe that there is no major effect on the results. In fact, as the real p-tau_rel_ values of controls are even lower than what we report, differences between groups would become larger.

Since Aβ_1–42_ and tau are related to AD pathology, it was expected that differentiation between non-AD dementias and controls would be less successful. Indeed, similar to the comparison between AD and FTLD, the tau-pathology seen in a subset of FTLD cases did not result in significant differences in either t-tau, p-tau_181_, or p-tau_rel_ levels between FTLD and controls. Overall, single markers and biomarker ratios achieved poor AUC values in this comparison, with the exception of the p-tau_181_/t-tau ratio (because the small differences in these markers were in opposite directions). Also for the differentiation between DLB and controls AUC values were poor, and no significant change was observed when p-tau_rel_ was added, although the Aβ_1–42_/p-tau_rel_ ratio had the highest raw AUC value. One successful differentiation was the diagnosis of CJD, with high AUC values for both t-tau and p-tau_rel_ alone as well as any ratio including t-tau or p-tau_rel_. This can again be explained by the fact that (non-phosphorylated) tau reflects the extensive neurodegeneration in CJD in the absence of tau-pathology [23].

### Study limitations: p-tau_rel_ biomarker values

This study was subject to at least two limitations. Firstly, the number of patients in each dementia group was quite small as we chose to select only patients with high diagnostic certainty (by either pathological confirmation or clinical follow-up). While this allowed for a clear separation between groups, it also put a limit on statistical analyses. Secondly, there was a high number of samples with p-tau_rel_ levels below the detection limit. This was especially the case in cohorts which typically also have relatively low t-tau values (controls, FTLD, DLB). As our results show p-tau_rel_ concentrations to be at least one sixth of t-tau levels (not seen in CJD because of the upper limit of detection in the t-tau assay) very low p-tau_rel_ values are not unexpected. It thus appears the assay used lacks the necessary sensitivity to detect true levels of p-tau_rel_, therefore precluding its potential use as biomarker in differential dementia diagnosis. Then again, the study by Lewczuk et al. found p-tau_rel_ concentrations from controls to fall within the detection range of the assay [10]. Going into detail, limited raw data in Lewczuk et al. [10] showed an average value of 109 ± 32.0 pg/mL p-tau_rel_ in AD/MCI and 62.1 ± 9.3 pg/mL in controls. In our study, the value for AD patients is very comparable (mean of 108 ± 13 pg/mL), while our control group had strikingly lower values with many p-tau_rel_ levels below the detection limit (mean of 39.4 ± 3.0 pg/mL). This difference is possibly related to control subject selection, but as there is only one other study to compare our results to, future independent research using the p-tau_rel_ assay will be necessary to interpret these discrepancies and to validate our results.

## Conclusions

In conclusion, this study shows that the addition of p-tau_rel_ to the AD CSF biomarker panel did not improve the differential diagnosis between AD and non-AD dementias. However, limited sensitivity of the assay might mask the potential diagnostic value of non-phosphorylated tau as a biomarker.

## Additional file

Additional file 1: Table S1. Correlations between different markers for tau. **Table S2.** Diagnostic performance of CSF biomarkers for differentiation between dementias and controls or differentiation between AD and non-AD dementias. (DOCX 28 kb)

## Abbreviations

AD: Alzheimer’s disease
AUC: Area under the curve
Aβ_1–42_: Amyloid-beta of 42 amino acids
CJD: Creutzfeldt-Jakob disease
CSF: Cerebrospinal fluid
CV: Coefficient of variation
DLB: Dementia with Lewy bodies
ELISA: Enzyme-linked immunosorbent assay
FTLD: Frontotemporal lobar degeneration
MCI: Mild cognitive impairment
MMSE: Mini-mental state examination
p-tau: Phosphorylated tau protein
p-tau_181_: Tau protein phosphorylated at threonine 181
p-tau_rel_: Non-phosphorylated tau fraction
ROC: Receiver operating characteristic
t-tau: Total tau protein
