# Supplementary material for: No added diagnostic value of non-phosphorylated tau fraction (p-taurel) in CSF as a biomarker for differential dementia diagnosis
Source: Alzheimers Res Ther. 2017 Jul 14;9:49. doi: 10.1186/s13195-017-0275-5 (PMC5513364; doi:10.1186/s13195-017-0275-5)
Supplement: Additional file 1: Table S1. — Correlations between different markers for tau. Table S2. Diagnostic performance of CSF biomarkers for differentiation between dementias and controls or differentiation between AD and non-AD dementias. (DOCX 28 kb) [file 13195_2017_275_MOESM1_ESM.docx]

**Additional file 1**

**Table S1. Correlations between different markers for tau.**

|  |  | p-tau_181_ | p-tau_rel_ |
| --- | --- | --- | --- |
| controls | t-tau | 0.70 (**p<0.001**) | 0.70 (**p<0.001**) |
|  | p-tau_181_ | / | 0.47 (**p=0.039**) |
| AD | t-tau | 0.89 (**p<0.001**) | 0.84 (**p<0.001**) |
|  | p-tau_181_ | / | 0.77 (**p<0.001**) |
| FTLD | t-tau | 0.77 (**p<0.001**) | 0.77 (**p<0.001**) |
|  | p-tau_181_ | / | 0.57 (**p<0.001**) |
| DLB | t-tau | 0.90 (**p<0.001**) | 0.70 (**p<0.001**) |
|  | p-tau_181_ | / | 0.65 (**p<0.001**) |
| CJD | t-tau | n.a. | n.a. |
|  | p-tau_181_ | / | 0.65 (**p=0.003**) |

Values are Spearman’s rho. All significances (p<0.05) are marked in bold. n.a. is not available.

**Table S2: Diagnostic performance of CSF biomarkers for differentiation between dementias and controls or differentiation between AD and non-AD dementias.**

|  | AD vs. controls | | | | |  | AD vs. non-AD | | | | |
| --- | --- | --- | --- | --- | --- | --- | --- | --- | --- | --- | --- |
|  | **AUC** |  | **cut-off** | **sens (%)** | **spec (%)** |  | **AUC** |  | **cut-off** | **sens (%)** | **spec (%)** |
| Aβ_1-42_ | 0.868 | ≤ | 719 | 95.6 | 75.0 |  | 0.642 | ≤ | 741 | 97.8 | 30.2 |
| t-tau | 0.926 | > | 401 | 84.4 | 85.0 |  | 0.734 | > | 412 | 84.4 | 64.2 |
| p-tau_181_ | 0.907 | > | 66.3 | 68.9 | 95.0 |  | 0.883 | > | 53.0 | 88.9 | 76.4 |
| p-tau_rel_ | 0.865 | > | 33.7 | 88.9 | 75.0 |  | 0.619 | > | 45.7 | 80.0 | 48.6 |
| Aβ_1-42_/t-tau | 0.976 | ≤ | 1.54 | 91.1 | 95.0 |  | 0.762 | ≤ | 1.30 | 88.9 | 60.4 |
| Aβ_1-42_/p-tau_181_ | 0.966 | ≤ | 7.28 | 80.0 | 100.0 |  | 0.860 | ≤ | 7.95 | 84.4 | 78.3 |
| Aβ_1-42_/p-tau_rel_ | 0.949 | ≤ | 15.6 | 95.6 | 80.0 |  | 0.657 | ≤ | 7.71 | 68.9 | 67.0 |
| p-tau_181_/t-tau | 0.812 | ≤ | 0.148 | 75.6 | 85.0 |  | 0.505 | > | 0.084 | 100.0 | 23.6 |
| p-tau_181_/p-tau_rel_ | 0.576 | ≤ | 1.00 | 53.3 | 75.0 |  | 0.652 | > | 0.85 | 68.9 | 58.3 |
|  |  |  |  |  |  |  |  |  |  |  |  |
|  | FTLD vs. controls | | | | |  | AD vs. FTLD | | | | |
|  | **AUC** |  | **cut-off** | **sens (%)** | **spec (%)** |  | **AUC** |  | **cut-off** | **sens (%)** | **spec (%)** |
| Aβ_1-42_ | 0.679 | ≤ | 708 | 60.5 | 75.0 |  | 0.689 | ≤ | 674 | 88.9 | 48.8 |
| t-tau | 0.622 | > | 272 | 67.4 | 60.0 |  | 0.862 | > | 412 | 84.4 | 74.4 |
| p-tau_181_ | 0.599 | ≤ | 32.5 | 37.2 | 85.0 |  | 0.933 | > | 49.0 | 93.3 | 81.4 |
| p-tau_rel_ | 0.588 | > | 72.1 | 22.0 | 100.0 |  | 0.799 | > | 45.7 | 80.0 | 68.3 |
| Aβ_1-42_/t-tau | 0.744 | ≤ | 2.05 | 53.5 | 85.0 |  | 0.893 | ≤ | 1.02 | 77.8 | 88.4 |
| Aβ_1-42_/p-tau_181_ | 0.573 | ≤ | 17.5 | 48.8 | 75.0 |  | 0.920 | ≤ | 7.95 | 84.4 | 90.7 |
| Aβ_1-42_/p-tau_rel_ | 0.711 | ≤ | 21.8 | 80.0 | 60.0 |  | 0.846 | ≤ | 7.71 | 68.9 | 85.0 |
| p-tau_181_/t-tau | 0.827 | ≤ | 0.153 | 83.7 | 80.0 |  | 0.571 | > | 0.122 | 60.0 | 58.1 |
| p-tau_181_/p-tau_rel_ | 0.695 | ≤ | 1.02 | 67.5 | 75.0 |  | 0.601 | > | 0.86 | 66.7 | 55.0 |

|  | DLB vs. controls | | | | |  | AD vs. DLB | | | | | |
| --- | --- | --- | --- | --- | --- | --- | --- | --- | --- | --- | --- | --- |
|  | **AUC** |  | **cut-off** | **sens (%)** | **spec (%)** |  | **AUC** |  | | **cut-off** | **sens (%)** | **spec (%)** |
| Aβ_1-42_ | 0.747 | ≤ | 714 | 73.3 | 75.0 |  | 0.637 | ≤ | | 686 | 91.1 | 31.1 |
| t-tau | 0.589 | > | 227 | 77.8 | 45.0 |  | 0.863 | > | | 377 | 88.9 | 73.3 |
| p-tau_181_ | 0.574 | > | 40.5 | 62.2 | 55.0 |  | 0.832 | > | | 53.0 | 88.9 | 68.9 |
| p-tau_rel_ | 0.717 | > | 33.7 | 68.9 | 75.0 |  | 0.706 | > | | 60.1 | 66.7 | 68.9 |
| Aβ_1-42_/t-tau | 0.730 | ≤ | 1.60 | 46.7 | 95.0 |  | 0.855 | ≤ | | 1.02 | 77.8 | 80.0 |
| Aβ_1-42_/p-tau_181_ | 0.739 | ≤ | 17.4 | 73.3 | 75.0 |  | 0.823 | ≤ | | 7.95 | 84.4 | 73.3 |
| Aβ_1-42_/p-tau_rel_ | 0.815 | ≤ | 16.8 | 82.2 | 75.0 |  | 0.747 | ≤ | | 7.71 | 68.9 | 77.8 |
| p-tau_181_/t-tau | 0.656 | ≤ | 0.162 | 57.8 | 75.0 |  | 0.757 | ≤ | | 0.149 | 77.8 | 68.9 |
| p-tau_181_/p-tau_rel_ | 0.666 | ≤ | 1.01 | 57.8 | 75.0 |  | 0.560 | > | | 1.19 | 42.2 | 80.0 |
|  |  |  |  |  |  |  |  |  | |  |  |  |
|  | CJD vs. controls | | | | |  | AD vs. CJD | | | | | |
|  | **AUC** |  | **cut-off** | **sens (%)** | **spec (%)** |  | **AUC** |  | **cut-off** | | **sens (%)** | **spec (%)** |
| Aβ_1-42_ | 0.778 | ≤ | 636 | 77.8 | 75.0 |  | 0.541 | ≤ | 220 | | 0.0 | 77.8 |
| t-tau | 0.978 | > | 545 | 94.4 | 100.0 |  | 0.896 | ≤ | 1170 | | 88.9 | 94.4 |
| p-tau_181_ | 0.503 | > | 42.0 | 55.6 | 60.0 |  | 0.894 | > | 54.7 | | 89.7 | 83.3 |
| p-tau_rel_ | 1.000 | > | 72.1 | 100.0 | 100.0 |  | 0.978 | ≤ | 274 | | 97.8 | 94.7 |
| Aβ_1-42_/t-tau | 1.000 | ≤ | 1.03 | 100.0 | 100.0 |  | 0.780 | > | 0.442 | | 77.8 | 72.2 |
| Aβ_1-42_/p-tau_181_ | 0.764 | ≤ | 19.2 | 77.8 | 65.0 |  | 0.809 | ≤ | 7.64 | | 82.2 | 72.2 |
| Aβ_1-42_/p-tau_rel_ | 1.000 | ≤ | 2.95 | 100.0 | 100.0 |  | 0.985 | > | 1.76 | | 95.6 | 94.1 |
| p-tau_181_/t-tau | 0.997 | ≤ | 0.069 | 100.0 | 95.0 |  | 1.000 | > | 0.069 | | 100.0 | 100.0 |
| p-tau_181_/p-tau_rel_ | 1.000 | ≤ | 0.113 | 100.0 | 100.0 |  | 1.000 | > | 0.113 | | 100.0 | 100.0 |

ROC curve analyses for single biomarkers and biomarker ratios are reported with area under the curve values (AUC) and cut-off at maximized Youden’s index, with corresponding sensitivity (sens) and specificity (spec).
